# Actigraphy, a valuable tool for objective sleep evaluation in psoriasis: a review

**DOI:** 10.1007/s00403-025-04187-x

**Published:** 2025-04-12

**Authors:** Katerina Vlami, Kleoniki Pantelidi, Maria Dalamaga, Evangelia Papadavid

**Affiliations:** 1 Second Department of Respiratory Medicine, Medical School, National and Kapodistrian University of Athens, Attikon General University Hospital, 1 Rimini street, Haidari, Athens 12462 Greece; 2 Second Department of Dermatology, Medical School, National and Kapodistrian University of Athens, Attikon General University Hospital, 1 Rimini street, Haidari, Athens 12462 Greece; 3 Department of Biological Chemistry, Medical School, National and Kapodistrian University of Athens, 75 Mikras Asias street, Athens, 11527 Greece

**Keywords:** Psoriasis, Actigraphy, Sleep disorders, Dermatology, Patient management

## Abstract

Psoriasis, a form of chronic inflammatory skin disease, shows wide variations in severity and comorbidities. Considering the relatively clear scientific evidence concerning its great physical and psychological effects, researchers began to use actigraphy as a scientific monitoring tool for its bidirectional relationship with sleep. To evaluate the usefulness of actigraphy as a means to diagnose sleep disturbances in psoriatic patients. We performed a systematic review using PubMed, Scopus and Google Scholar to look at the use of actigraphy in research into psoriasis and sleep disorders. This review covered publications through February 2024. Included records were primary and secondary research papers. Actigraphy consistently revealed strong links between pruritus nighttime arousal, and poorer subjective sleep, although next-day psoriasis symptoms did not always worsen. Systematic analyses emphasized actigraphy’s role in measuring nocturnal scratching, supported by emerging smartwatch apps. Psoriatic arthritis studies likewise reported significant sleep disruptions, though evidence remains limited. From these studies, actigraphy presented as a useful tool in both clinical and research contexts, for diagnosing and monitoring sleep disorders among psoriatic patients. Actigraphy is an important test for psoriatic sleep disorders, providing useful objective data. This way the bridge between psoriasis and sleep be clearly understood, and treatment methods improved.

## Introduction

Psoriasis is a particularly complex form of chronic inflammatory skin disease. It ranges in severity from a few scattered red, scaly plaques to almost the entire body surface being affected. The prevalence of the disease ranges from 0.51 to 11.43% in adults [1]. Its prevalence is very different among populations: relatively low in Asian and higher among Caucasian and Scandinavian populations [2]. Psoriasis vulgaris is the most common form of psoriasis, and it has clinical subtypes with differing characteristics [3]. The effects of the disease are not limited to skin. Its effects also cover significant physical and psychological issues, such as psoriatic arthritis, inflammatory bowel disease, obesity, diabetes, cardiovascular diseases, obstructive sleep apnea, depression and anxiety [4]. All these comorbidities lead to reduced health-related quality of life and an increased disease burden [4].

Insomnia is common among patients with psoriasis; a bidirectional relationship is shared between them. Many factors make people with psoriasis prone to sleeping worse. Pruritus is a very common symptom of psoriasis, which usually exacerbates at night, which interferes with falling asleep and maintaining sleep. Pain, burning, or sensitivity in the skin may also add to discomfort in bed. An individual who has psoriasis has a greater susceptibility to anxiety, depression, and stress, all elements that could potentially interfere with patterns of sleep. The irritation of handling a condition that is chronic can lead to ruminating thoughts and difficulty unwinding at bedtime. Psoriasis is a chronic inflammatory disease, and systemic inflammation has effects on the regulation of sleep. Increased levels of cytokines such as TNF-alpha and IL-17 in psoriasis may be contributors to poor quality of sleep. Psoriasis is associated with a number of conditions which can also affect sleep such as obstructive sleep apnea and metabolic syndrome.

Insomnia is a core characteristic of psoriasis [5]. Complaints include inability to fall asleep properly and inability to stay asleep [5]. Such individuals are even more apt to suffer from wakes or exacerbations during the night [5, 6]. There are psycho-physiological links between psoriasis and sleeplessness, both directly (psoriasis is accompanied by itching) and indirectly [7]. Further studies of sleep problems using semi-structured interviews have provided a rich understanding of the ways in which psoriasis-related worries disturb sleep and vice versa [8]. The bi-directional nature of this relationship highlights the connection between psoriasis and sleep quality [8].

In exploring this relationship, actigraphy emerges as a valuable tool. Using a device to record and analyze movement, the information can be stored as an objective indicator of sleep patterns [9]. Actigraphy is a technique used for monitoring rest and activity cycles-very minimally invasive. Actigraphy is typically carried out for eliciting sleep patterns wherein an individual wears a small wrist-worn device known as an actigraph sensor for some days or weeks to record movements. The device keeps recording movements all through, thus providing data on periods of activity and rest. Software then processes this data to derive estimates for sleep onset, duration, efficiency, and wake times. Despite its limitations in diagnosing specific sleep disorders, actigraphy has proven useful in studying group differences, variations in sleep patterns over time, and the effects of behavioral or treatment interventions in both control individuals and certain patient groups [9].

Psoriasis and sleep disturbance are closely related, though the precise nature of their relationship is complex and not fully understood. Actigraphy, with its ability to monitor rest/activity cycles non-invasively, offers a valuable perspective in the assessment of sleep quality, duration, and patterns [9, 10]. The purpose of actigraphy is to record long periods of time sleeping in the real world, so as to get a better understanding of the actual disturbances that happen at night among psoriatic patients. In this review we are concerned with the use of actigraphy to provide an objective measure of sleep disturbances among psoriatic patients. We present a comprehensive examination of the literature relating to the application of actigraphy in research into psoriasis-related sleep disruption. We aim to explore how checking the connection between sleep parameters measured with actigraphy and objective indicators of factors related to psoriasis such as disease severity, response to treatment or quality of life can help us understand and improve disease management. Managing sleep disturbances is an integral part of holistic psoriasis management, as treating them improves patient QoL. To the best of our knowledge, this is the first review to evaluate the use of actigraphy in psoriasis. Therefore, the aim of this review is to.

1. Present the potential of actigraphy to monitor changes in sleep structure, including nocturnal scratching, among psoriatic patients.
2. Discuss how improved sleep management, as guided by actigraphy findings, can positively impact the overall quality of life for patients with psoriasis.
3. Provide a comprehensive overview of current research and findings on the use of actigraphy in the context of psoriasis and sleep disturbances.
4. Summarize the key insights gained from actigraphy-based research about the impact of psoriasis on sleep patterns and vice versa.

## Methods

**Fig. 1:**
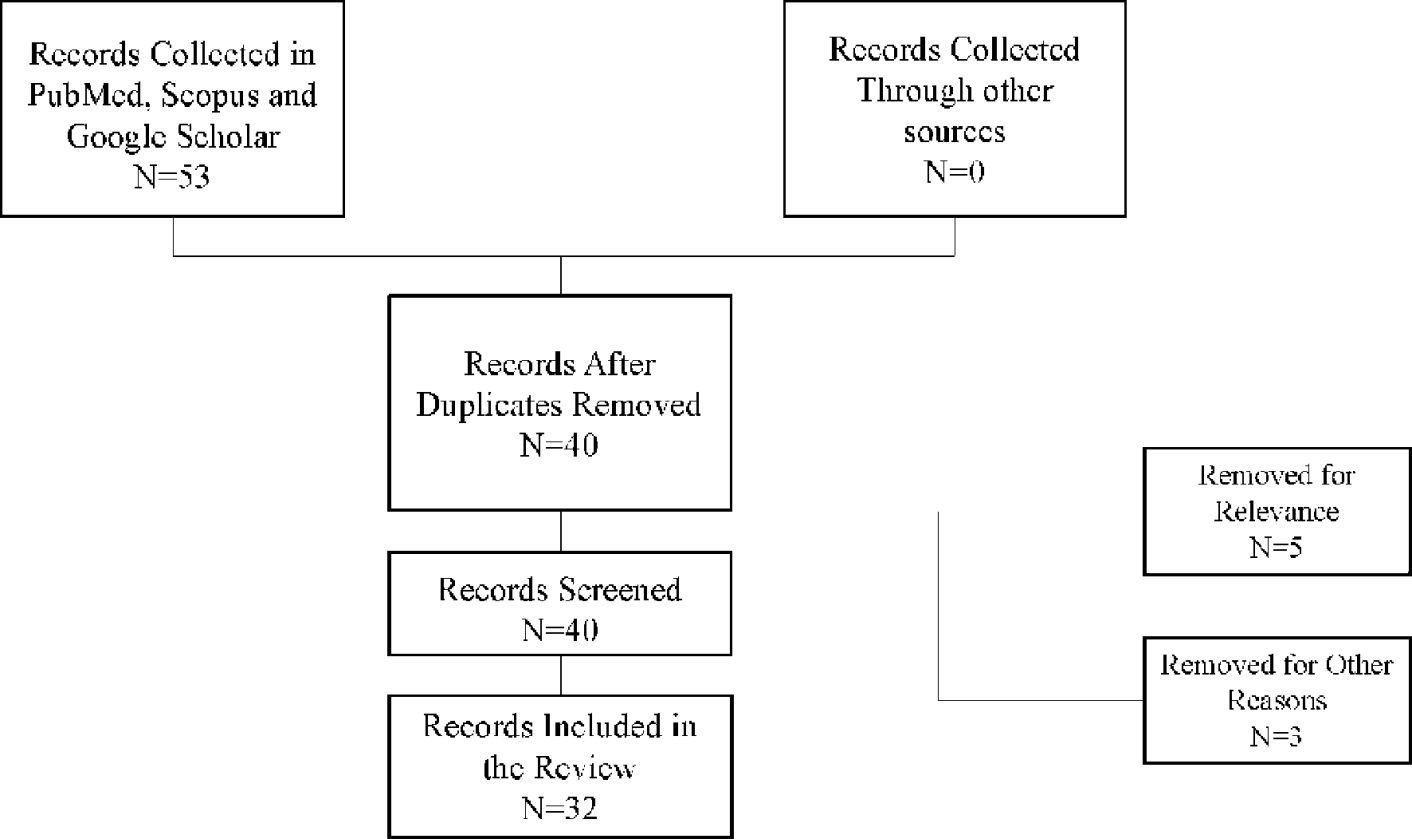
Record screening criteria

In this review, a detailed systematic literature search was conducted, focusing on the intersection of sleep disorders and psoriasis as evaluated by actigraphy. The search was conducted on the electronic databases PubMed, Scopus and Google Scholar. We employed an advanced search strategy incorporating Medical Subject Headings (MESH) terms. Our primary search queries were structured as follows: (“psoriasis” OR “psoriatic skin conditions”) AND (“sleep disorders” OR “sleep disturbances”) AND (“actigraphy” OR “movement monitoring”) and modified to the parameters of each database. We then screened articles at the title/abstract level, and full texts were assessed if deemed relevant to psoriasis, sleep, and actigraphy. In addition, we scanned the reference lists of retrieved articles to identify further relevant works. This strategy was designed to capture a comprehensive range of research, targeting how sleep disturbances are associated with the phenotype and QoL in psoriatic patients.

We included primary (original) research studies, observational or interventional, reporting any actigraphy-based measurement in psoriasis. We also included secondary (review or meta-analysis) articles that addressed both psoriasis and sleep with specific mention of actigraphy or objective measurement tools. Exclusion criteria comprised non-dermatologic studies, duplicate records, conference abstracts without any actigraphy data, and articles with incomplete methodology.

Based on the final pool of included studies, actigraphy monitoring periods ranged from 5 days to 14 days for the majority of prospective cohorts, with some studies extending to 2 or more weeks. In atopic dermatitis and psoriasis research alike, 7–14 days of continuous actigraphic recording was the most commonly reported protocol.

## Results

### Study characteristics

Our title and abstract screening yielded a total of 53 original articles exploring psoriasis, actigraphy, and the relationship between them. From these, we identified and removed 13 duplicates, which streamlined our review process. Further evaluation led to the exclusion of 7 records due to irrelevance to our specific topic of interest and 4 articles for other reasons (generalization, lack of validity). Consequently, we were left with 32 pertinent records, which formed the backbone of this comprehensive review (Fig. 1). Of these 32 papers, 6 were identified as key, in-depth records specifically focusing on actigraphy-based findings in psoriasis or highly comparable dermatologic conditions (see Table 1). The articles chosen spanned a publication timeline from 2001 to 2024, reflecting over two decades of evolving research in this domain. The selected studies encompassed a diverse array of research types, including other literature reviews, clinical studies, empirical research studies and observational analyses. Notably, the studies varied in their approach and focus, ranging from clinical assessments of sleep quality in psoriasis patients to more technical evaluations of actigraphy as a tool for monitoring nocturnal activities like scratching. Primary studies typically examined either the correlation between psoriasis severity and sleep metrics, or the utility of wrist devices in detecting pruritus-induced awakenings.

Across the primary studies included in this review, the mean age of psoriasis participants ranged from the late 30s to mid-50s, with female representation typically around 50–60%. Overall, the sex distribution showed a relatively balanced male-female ratio, with a slight female preponderance in some samples. Age tended to vary but included mostly middle-aged adults across the board.

### Insights from prospective studies

In a landmark prospective study leveraging actigraphy and experience sampling methodology, researchers discovered pivotal insights into the nocturnal experiences of individuals with psoriasis [11]. The study highlighted a complex interplay between increased nighttime arousal, tracked via actigraphic data, and the deterioration of self-reported sleep quality [12]. This research pointed to a significant absence of correlation between objective sleep parameters and daytime psoriasis symptoms, showing the intricate and perhaps indirect ways psoriasis impacts nocturnal rest. Interestingly, higher rates of night-time sleep arousal (as measured by the Sleep Interference Rating Scale) were associated with worse self-reported TST and SE and poorer self-reported sleep quality but not with objective measurements [11]. The significance of this paradoxical discovery is that in the relationship between psoriasis and sleep responses, these two variables could be connected by indirect and multiple mechanisms [5, 11, 13]. In addition, the study also found that sleep parameters-whether measured objectively or subjectively-were not linked to next-day psoriasis symptoms. On the other hand, there was a clear link between perceived sleep and nighttime wakings. In particular, those disturbances in the middle of the night were correlated with lower self-reported sleep quality (in TST, SE and sleep quality) but psoriasis symptoms were not linked to night-time sleep, and no deterioration of the condition occurred after poor sleep [11]. From this, we surmise that the effects of disturbed sleep on psoriasis are probably not as simple as previously believed [11].

### Systematic analyses

Systematic literature reviews, encompassing a spectrum of dermatological conditions including psoriasis, have highlighted the indispensability of objective sleep evaluation tools like actigraphy in understanding sleep disturbances [14]. Detailed reports conclude that there is a high incidence of sleeping disorders in all these conditions [14–16]. They note that accurate evaluation and control of the effects these conditions have on sleep quality require strict, quantifiable standards. Research has shown nocturnal pruritus (NP) to be a type of dermatologic complaint that often causes sleep deprivation and poor quality of life, reduces physical and mental health, and can even worsen negative emotional states [17–19]. Its pathophysiology and effects on sleep patterns and quality have only been partially elucidated, with possible causes including impaired skin barrier function and changes in serum levels of endogenous substances (e.g., cortisol) [20]. Probably the most important function of actigraphy here is to provide an objective tool with the added benefit of quantitative data to support subjective judgements of sleep quality, which in the past have dominated most dermatologic research into sleep [10, 20]. This dual subjective-objective examination is very helpful when seeking to gain a broad view of sleep disturbance in dermatological diseases, as well as to fill the gap created by many quality of life instruments for skin disorders that exclude objective tools out of their assessment [14].

### Technological innovations in measurement

Research into psoriasis is undergoing a qualitative change with the advent of technologies for measuring changes in real time [21]. Continuing research has begun to develop smartwatch applications that can accurately track nocturnal scratching [22, 23]. This type of technology, best illustrated by the Itch Tracker app [24], has proven to be accurate at detecting scratching episodes-a commonly seen symptom in psoriasis sufferers. In another study using patients with atopic dermatitis, the app was proven to have a positive predictive value of 90.2 ± 6.6% and sensitivity of 84.6 ± 10.2%, making it suitable for clinical use as an alternative to video monitoring [21, 24].

Moreover, the positive correlation between scratching duration and their Eczema Area and Severity Index (EASI) score validates the app’s applicability in practice [21]. All of these technological advances complement and enhance current actigraphic measurements, opening up a new dimension of personalized patient monitoring. Such technology can be integrated into clinical practice, thereby greatly enhancing our awareness of the nocturnal symptoms associated with a variety of dermatological disorders such as psoriasis; ultimately this provides a better foundation for their treatment and care [24].

**Table 1:** Overview of the six most important records

Study Reference		Focus Area	Key Findings	Methodological Notes
(Henry et al., 2020)	[11]	Psoriasis and Sleep Disturbance	Link between night-time arousal and poor sleep quality; no direct correlation with daytime symptoms.	Prospective study integrating actigraphy and experience sampling.
(Sandoval et al., 2014)	[25]	Comparative Dermatological Research	Positive correlation between atopic dermatitis severity and sleep disturbances.	Utilization of actigraphy in assessing atopic dermatitis.
(Podder et al., 2021)	[14]	Systematic Reviews on Dermatological Conditions	Actigraphy highlighted as a key tool in understanding sleep disturbances across dermatological diseases.	Broad literature review including psoriasis.
(Ikoma et al., 2019)	[24]	Innovative Measurement Techniques	High efficacy of smartwatch apps in detecting nocturnal scratching.	Technological advancement in actigraphy-like measurements.
(Gowda et al., 2010)	[26]	Sleep Quality and Psoriasis Severity	Correlation between sleep disturbance and psoriasis severity.	Actigraphy used to quantify sleep quality in relation to disease severity.
(Grant et al., 2023)	[13]	Psoriatic Arthritis and Sleep	High prevalence of sleep problems in PsA patients, as measured by actigraphy.	Extending actigraphy’s application to PsA.

These six records collectively provide the clearest evidence and most detailed insights into the value of actigraphy for understanding psoriasis-related sleep disturbance. Henry et al. (2020) integrates actigraphy with experience sampling to explore nighttime arousal and daily functioning in psoriasis, offering a nuanced view of why poor sleep does not always exacerbate next-day psoriatic symptoms. Sandoval et al. (2014), though focused on atopic dermatitis, was among the first to link treatment-induced changes in skin condition to objectively measured sleep parameters, illustrating how actigraphy can capture nuances missed by subjective reports. Podder et al. (2021) presents a systematic review revealing widespread nocturnal pruritus in dermatologic disorders, reinforcing actigraphy’s importance in substantiating sleep complaints. Ikoma et al. (2019) introduces an innovative smartwatch-based scratch-detection method, hinting at the potential for user-friendly actigraphy-like solutions. Gowda et al. (2010) synthesizes how psoriasis-driven pruritus, depression, and even obstructive sleep apnea form a vicious cycle of poor sleep, while Grant et al. (2023) highlights the underexamined but significant burden of sleep disruption in psoriatic arthritis. Collectively, they illustrate how objective tools like actigraphy broaden our understanding of the complex, bidirectional nature of psoriasis and sleep.

### The interplay between sleep quality and disease severity

Multiple actigraphic assessments, which use motion sensors to monitor sleep architecture, have demonstrated a positive correlation between the severity of psoriasis and disruption of sleep. This indicates a complex relationship in which psoriasis interferes with sleep as well as being exacerbated by poor sleep [27]. We noticed a high correlation among psoriasis sufferers between factors such as pruritus, depression, pain and even obstructive sleep apnea and loss of sleep [26]. For instance, data collected through actigraphy show that awakenings and sleep fragmentation caused by pruritus directly negatively affect overall sleep quality. Psoriasis severity, as well as comorbidity severity assessment and diagnosis, can be assisted through an actigraphic analysis of sleep pattern disturbances [17, 20, 24, 27]. Furthermore, pain from psoriatic lesions and obstructive sleep apnea (both more common among psoriatic patients [26]) can be measured quantitatively using actigraphy to gauge their impact on the patient’s sleep [22]. The reasons behind these synergies involve the fact that they all share common inflammatory pathways [28]. Hence, actigraphy is an important common denominator that bridges the processes of evaluation and treatment [26].

### Extending actigraphy to psoriatic arthritis

While most actigraphy research focuses on chronic plaque psoriasis, psoriatic arthritis (PsA) also harbors a significant burden of sleep disturbance [13]. PsA patients commonly report insomnia, pain-related awakenings, and fatigue—all potentially measurable via actigraphy. However, objective sleep studies (actigraphy or PSG) in PsA remain sparse, and more research is needed to validate self-reported measures specifically in PsA populations. Future research should validate self-reported sleep measures in patients with psoriatic arthritis, use both objective and subjective sleep measures to examine how sleep quality is associated with disease activity and psoriatic arthritis symptoms, and evaluate the effectiveness of strategies to treat sleep problems and the effects of such treatments on symptoms and signs in patients with psoriatic arthritis. In the realm of psoriatic arthritis (PsA), actigraphy has emerged as a critical tool in objectively quantifying sleep disturbances. Studies in this domain reveal a concerning prevalence of sleep problems among PsA patients [13].

## Discussion

### Objective short sleep duration in psoriasis

In the realm of sleep disorders, actigraphy has emerged as a pivotal tool for diagnosing objective short sleep duration insomnia, notably identified as the most biologically severe phenotype of insomnia [29]. This distinction is crucial as it is intimately linked with heightened biological severity and an array of medical complications, including hypertension, diabetes, and an increased mortality risk. Crucially, objective short sleep duration serves as a biomarker for genetic predisposition to chronic insomnia.

The relevance of this finding extends to patients with psoriasis, a group shown to predominantly exhibit this severe insomnia phenotype, as evidenced through actigraphic assessments [30]. This intersection between psoriasis and severe insomnia phenotype underscores a broader pattern of sleep disturbances within this patient population. Notably, despite systemic treatment for psoriasis, the persistence of disrupted sleep patterns highlights the need for targeted insomnia interventions [30].

Moreover, psoriasis is tightly connected with Obstructive Sleep Apnea (OSA). This link hints a possible bidirectional relationship [31, 32]. It suggests a more complicated connection between psoriasis and sleeping problems, needing a more holistic approach to handle it.

### Main takeaways

From the studies reviewed, a consistent theme emerges: actigraphy serves as a powerful, minimally invasive tool to capture changes in sleep architecture among individuals living with psoriasis. Multiple investigations have demonstrated that pruritus, mood disturbances, and systemic inflammation can negatively affect total sleep time and sleep efficiency, yet the link between poor sleep and disease severity is not always linear. For example, heightened nighttime arousal correlates with poor subjective sleep, but does not always translate into next-day exacerbations of psoriasis [11]. This complexity presents the need for both objective and subjective measures to fully characterize sleep-wake dynamics.

Although polysomnography (PSG) remains the gold standard for diagnosing sleep disorders such as OSA, it is expensive and limited to clinical settings. Actigraphy, by contrast, can be performed at home over extended periods, capturing fluctuations in real-world conditions. This approach is essential given that patients with psoriasis often experience variations in symptoms and pruritus from night to night. When insomnia is suspected, combining actigraphic monitoring with validated patient-reported measures offers a balanced way to identify triggers—such as stress and inflammation—and tailor management strategies accordingly. Dermatologists and sleep specialists can employ actigraphy to identify patient-specific triggers of nocturnal itch or arousals, track changes in sleep parameters over time or in response to systemic therapies, and correlate daily variations in itch or pain diaries with objective movement data. Such an approach provides valuable insights into the patient’s routine, enabling more precise and timely interventions.

Psoriatic arthritis extends this discussion further. Sleep problems are pervasive in psoriatic arthritis, yet large-scale investigations employing actigraphy are relatively scarce [13]. Enhanced understanding of how joint pain, immune dysregulation, and fatigue converge at night could yield more refined, personalized interventions. Meanwhile, advances in digital health, signal that specialized smartwatch apps may soon augment or even replace standard actigraphy, allowing for broader accessibility and real-time data feedback [24].

Overall, the evidence indicates that objective measures of sleep can offer clinicians a fuller picture of psoriasis severity and treatment response. By integrating actigraphy into routine care, alongside patient-centered assessments, physicians may identify the root causes of nighttime discomfort and design more targeted therapeutic approaches to improve both skin health and sleep quality.

### Limitations and future directions

Variation in actigraphy devices, monitoring durations (commonly 5–14 days), and scoring algorithms continues to complicate direct comparisons across studies. Psoriatic arthritis, while recognized as a significant comorbidity, remains underrepresented in the literature, highlighting the need for dedicated investigations using actigraphy in PsA populations. Furthermore, the growing availability of pruritus-specific apps (e.g., “Itch Tracker”) demonstrates promise but also underscores the lack of standardized validation protocols. Integrating biological markers, such as IL-17 and CRP, with actigraphic outcomes could offer a clearer picture of the inflammatory-sleep axis and pave the way for more targeted therapeutic interventions.

## Conclusion

Actigraphy and polysomnography (PSG) are valuable tools for assessing sleep, but they serve different purposes and are appropriate for different situations:

- Accuracy and detail: Polysomnography is the gold standard for diagnosing sleep disorders because it provides comprehensive measurements of brain activity, eye movements, muscle tone, and heart rhythm. Although less detailed, actigraphy provides reliable estimates of sleep patterns over time, making it useful for tracking sleep habits and circadian rhythms.
- Convenience and cost: Actigraphy is non-invasive, portable, and inexpensive, making it ideal for long-term, real-world sleep monitoring. In contrast, PSG is more expensive and is typically performed in a clinical setting, limiting its use.
- Applications: PSG is essential for diagnosing conditions such as sleep apnea, REM sleep behavior disorder, and narcolepsy. On the other hand, actigraphy is better suited for monitoring sleep patterns in patients with insomnia, circadian rhythm disorders, or for research purposes.

In summary, PSG provides detailed diagnostic information, while actigraphy provides convenient, long-term sleep monitoring. The choice between the two depends on the specific clinical or research needs. Often, combining both approaches can provide the most comprehensive understanding of sleep health.

The main conclusion of this comprehensive review is the key role of actigraphy in improving our understanding of psoriasis-related sleep disturbances. Using non-invasive, objective tools such as actigraphy to observe the sleeping posture and sleep quality of people with psoriasis can provide valuable insights into their condition. This closes the gap between subjective and objective observations and provides more accurate information about nighttime behaviors.

Studies have shown that actigraphy can accurately record the interaction between psoriasis severity and sleep deprivation. This allows for quantification of nighttime awakenings, the extent of sleep disruption, and changes in overall sleep architecture. These are factors that have been associated with psoriasis symptoms such as itch, depression, and pain. This objective data will be of great benefit to those trying to understand the interrelationship between sleep disturbances and the physical and mental burden of psoriasis.

In essence, actigraphy can be a useful tool in the comprehensive treatment of psoriasis. By advancing the understanding of the relationship between psoriasis and sleep, actigraphy can help doctors develop personalized solutions that can provide patients with better options for managing their skin disease symptoms and sleep disturbances.

## Data Availability

No datasets were generated or analysed during the current study.
